# The Role of Structural Elements of the 5'-Terminal Region of p53 mRNA in Translation under Stress Conditions Assayed by the Antisense Oligonucleotide Approach

**DOI:** 10.1371/journal.pone.0141676

**Published:** 2015-10-29

**Authors:** Agata Swiatkowska, Paulina Zydowicz, Agnieszka Gorska, Julia Suchacka, Mariola Dutkiewicz, Jerzy Ciesiołka

**Affiliations:** Institute of Bioorganic Chemistry, Polish Academy of Sciences, Noskowskiego 12/14, 61-704, Poznan, Poland; Universidad Nacional Autonoma de Mexico, MEXICO

## Abstract

The p53 protein is one of the major factors responsible for cell cycle regulation and stress response. In the 5’-terminal region of p53 mRNA, an IRES element has been found which takes part in the translational regulation of p53 expression. Two characteristic hairpin motifs are present in this mRNA region: G56-C169, with the first AUG codon, and U180-A218, which interacts with the Hdm2 protein (human homolog of mouse double minute 2 protein). 2′-*O*Me modified antisense oligomers hybridizing to the 5'-terminal region of p53 mRNA were applied to assess the role of these structural elements in translation initiation under conditions of cellular stress. Structural changes in the RNA target occurring upon oligomers’ binding were monitored by the Pb^2+^-induced cleavage method. The impact of antisense oligomers on the synthesis of two proteins, the full-length p53 and its isoform Δ40p53, was analysed in HT-29, MCF-7 and HepG2 cells, under normal conditions and under stress, as well as *in vitro* conditions. The results revealed that the hairpin U180-A218 and adjacent single-stranded region A219-A228 were predominantly responsible for high efficacy of IRES-mediated translation in the presence of stress factors. These motifs play a role of *cis*-acting elements which are able to modulate IRES activity, likely *via* interactions with protein factors.

## Introduction

The tumour suppressor protein p53 is one of the major transcriptional regulators in the cell, particularly under stress conditions [1]. In the presence of various stress factors, p53 is activated *via* post-translational modifications and the expression pattern of several genes is changed to maintain genetic stability and prevent carcinogenesis [2–4]. Under normal conditions, the level of p53 in the cell is kept low, mostly due to rapid protein degradation by the E3 ubiquitin-ligase activity of Hdm2 [5].

The *p53* gene is widely controlled at each expression level. Moreover, as a consequence of various transcription initiation sites, alternative splicing, and alternative sites of translation initiation, several isoforms of p53 protein are synthetized in the cell [1, 6, 7]. In recent years, new data has suggested that these isoforms play an important role in the regulation of p53 activity in the cell, tumour transformation, in the cellular response to bacterial and viral infections, and to stress conditions [8–10]. Usually, besides full-length p53 (FLp53) the most abundant isoform in the cell is the Δ40p53 isoform, which lacks the first 39 amino acids of p53 sequence of the N terminus. The Δ40p53 is able to oligomerize with FLp53 [11, 12] thus reducing the total pool of free FLp53, and possibly, due to the presence of the DNA binding domain, competing with FLp53 in gene regulation [13]. The lack of the Hdm2 (human homolog of mouse double minute 2 protein) binding site in Δ40p53 prevents this isoform from undergoing Hdm2 regulation; consequently, it might not be involved in the stress response pathway in the same manner as FLp53 [14]. Recently, it has been shown that endoplasmic reticulum stress promotes PERK-dependent induction of Δ40p53 translation and its homo-oligomerization [15]. This isoform is significantly upregulated in breast tumour tissue [16].

It is thought that the synthesis of FLp53 and Δ40p53 is regulated at the translational level. In the 5'-terminus of p53 mRNA an IRES (Internal Ribosomal Entry Site) element has been identified. Several proteins interacting with this region seem to play the role of ITAFs (IRES-*trans* acting factors) [14, 17, 18]. IRES-dependent translation of FLp53 and Δ40p53 is mostly triggered during cellular stress, apoptosis and phase transition when cap-dependent process is impaired. Under different stress conditions, when IRES-mediated translation occurs, changes in the p53/Δ40p53 ratio have been observed [18].

**Fig 1:**
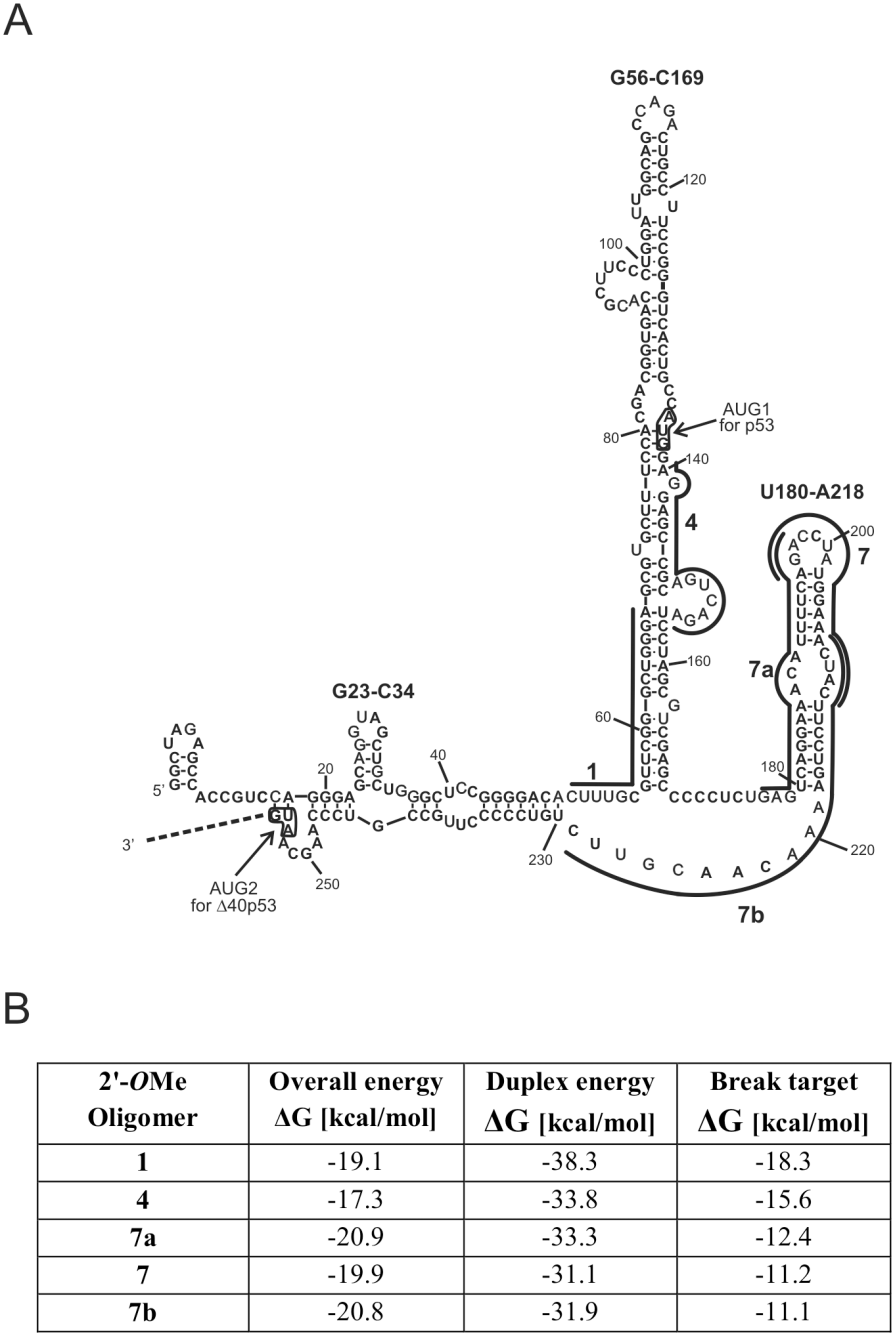
The 5'-terminal region of p53 mRNA and complementary antisense oligonucleotides. **(A)** The antisense oligomer binding sites are displayed in this secondary structure model of the 5'-terminal region of p53 mRNA [19, 27]. **(B)** Energy requirements for RNA target-oligomer hybrid formation calculated by the OligoWalk function in the RNAstructure 5.6 program [30]. Overall ΔG—the target-oligomer binding, including breaking RNA target structure and oligomer self-structure; duplex ΔG—the target-oligomer binding from unstructured states; break target ΔG—the energy penalty due to breaking of intramolecular RNA target base pairs when the oligomer is bound.

Recently, we have proposed a secondary structure model of the 5'-terminus of p53 mRNA containing IRES [19]. It turns out that in folding of the 5' non-coding part of p53 mRNA p53 the coding sequence up to the second initiation codon for Δ40p53 is involved (Fig 1). Two characteristic hairpin motifs are present in the secondary structure model of this mRNA region, one hairpin containing the first AUG codon, and the other hairpin, which has been demonstrated to interact with Hdm2 [20]. Despite the expanding knowledge about the p53 and Δ40p53 translation processes, little is known about the correlation between the structure of putative the IRES and the efficiency of p53 synthesis. Extensive work on viral IRESs has shown a strong correlation between structural organization of these elements and their ability to interact with cellular ITAFs and to recruit the ribosome [21, 22]. The p53 IRES can also employ many cellular protein factors, likely acting as ITAFs, to regulate the translation of p53 and its isoforms [20, 23–25]. Since well-defined RNA structural motifs are present in the p53 mRNA region containing IRES, a question arises regarding whether these motifs must be preserved to maintain IRES activity. Alternatively, structural flexibility might be permissible, or even necessary, in this mRNA region under specific circumstances. Recently, it has been suggested that p53 IRES can assume different conformations during synthesis of p53 mRNA and that the HDMX protein acts as an RNA chaperone to fold this region into a functional structure [26].

The aim of our work was to assess the role of structural motifs present in the 5'-terminal region of p53 mRNA in the translation initiation process occurring upon stress conditions by means of the antisense oligonucleotide approach. We have previously successfully used antisense oligomers to modulate FLp53 and Δ40p53 expression at the RNA and protein levels [27]. Recently, an antisense strategy was also successfully used in p53 mRNA splicing alterations [28]. In our current study we used oligomers hybridizing to the 5'-terminus of p53 mRNA to partly rearrange the p53 IRES structure. Since the oligomers were entirely 2′-*O*Me modified, they could not activate RNase H and thereby trigger mRNA cleavage. In contrast, they could only act by inducing RNA misfolding or sterically hindering the translating ribosome [29]. Such perturbation of the translation process was also expected to change the levels of FLp53 and Δ40p53 synthesis. It might indicate which structural elements of the p53 IRES are crucial for its activity in translation of each protein in the presence of stress factors. To demonstrate whether possible correlation between the IRES structure and its function is similar regardless of the cell type and the p53 status, antisense oligomers were applied in various cell lines. The HT-29, MCF-7 and HepG2 cells were used which are characterized by different p53 and Δ40p53 expression levels.

## Materials and Methods

### DNA template constructs and oligonucleotides

The DNA templates for the 5'-terminal region of p53 mRNA, p53-554 RNA and Hp53-554 RNA were prepared as described previously [19]. Modified 2′-*O*Me oligomers No. 1, 4, 7, 7a and 7b, as well as the control oligomer were synthesized by Future Synthesis, Poznan, Poland. The sequences of these oligomers are shown in Table 1. DNA oligomers R554AUGb and Rdlug were purchased from Oligo Service IBB PAS, Warsaw.

**Table 1:** 2′-*O*Me antisense oligonucleotides.

2′-*O*Me Oligomer	Length [nt]	Sequence (5'-3')	Target site in p53 mRNA (5'-3')
c	21	ACCAGGGCGTATCTCTCCATA	*Firefly* luciferase coding sequence
1	19	TCCCAGCCCGAACGCAAAG	50–68
4	17	TCCTCGGCGTCAGTCTA	140–156
7a	21	TCTGAAAATGTTTCCTGACTC	177–197
7	19	GAAGTAGTTTCCATAGGTC	196–214
7b	21	AACGTTGTTTTCAGGAAGTAG	208–228

### Cell culture, antisense oligonucleotide transfection and stress conditions

HT-29 cells (originally from ECACC) were a gift from Dr M. Swiatkowska from the Medical University of Lodz, MCF-7 cells (originally from ECACC) were a gift from Prof. W. Krzyzosiak from IBCh PAS in Poznan, HepG2 cells were purchased from ECACC. HT-29, MCF-7 and HepG2 cells were maintained in McCoy’s, DMEM and MEM medium, respectively. All mediums were supplemented with 10% fetal bovine serum, non-essential amino acids (Gibco-BRL), 100 U/ml of penicillin G, 0.1 mg/ml of streptomycin sulphate (Sigma) and cells were kept at 37°C in 5% carbon dioxide atmosphere. Cells between passages 4 and 20 were used for transfection. Transfection was performed when cell confluence reached 50–80%. 2′-*O*Me antisense oligomers at a final concentration of 1 μM were transfected into cells using Lipofectamine RNAiMax according to the manufacturer’s transfection protocol (Invitrogen). The transfected cells were washed with PBS and given fresh medium 4 hours after transfection was initiated. Oxidative stress was generated by addition of hydrogen peroxide to a final concentration of 100 μM or 200 μM. Etoposide, freshly dissolved in DMSO, was applied at a final concentration of 50 μM. Cells were exposed to stress conditions for 6 hours, then harvested.

### Western blot

For Western blots, cell lysates were prepared in the buffer: 62.5 mM Tris-HCl pH 6.8, 2% SDS, 10% glycerol, 50 mM DTT and protease inhibitor (Roche). Total cell lysates were incubated for 5 min at 95°C and then loaded on a 10% SDS-PAGE gel and proteins were transferred to a nitrocellulose membrane. The blot was probed with mouse monoclonal antibodies: p53 (Pab 1801 and DO1), Hdm2 (SMP14) and GAPDH (A-3) (Santa Cruz Biotechnology). Primary antibody was detected by Goat Anti-Mouse-HRP (Thermo Scientific Pierce) and visualized by using the enhanced chemiluminescent visualization (ECL) system (Thermo Scientific Pierce).

### RNA isolation and RT-PCR

For RT-PCR, after transfection of HT-29, MCF-7 and HepG2 cells with antisense oligonucleotides and incubation in the absence or presence of hydrogen peroxide at a final concentration of 100/200 μM, total RNA was isolated from the cells using TriReagent (Molecular Research Centre, Inc.) according to the manufacturer’s protocol. RT-PCR was performed as previously described [27]. Briefly, the cDNA was prepared from 300 ng of RNA using 100 ng of oligo(dT)_18_ primer and 100 units of SuperScript^TM^ III reverse transcriptase (Invitrogen). Equal volumes of cDNA were used to amplify p53 and ß-actin using the following primers: p53 F, 5′-CTAGAGCCACCGTCCAGGGAGC-3′, p53 R, 5′-GTCTTGGCCAGTTGGCAAAACATC-3′, ß-actin F, 5′-AGAGCAAGAGAGGCATCCTG-3′, ß-actin R, 5′-CGACGTAGCACAGCTTCTCC-3′.

### *In vitro* transcription

*In vitro* transcription of p53-554 RNA corresponding to 554 nucleotides from the 5'-terminus of p53 mRNA and Hp53-554 RNA with an additional sequence of a stable hairpin at the 5' end of the template was performed as previously described [19]. Transcription reaction with AmpliScribe T7, T3 and SP6 High Yield Transcription Kit (Epicentre Biotechnologies) was performed as recommended by the manufacture’s protocol. After the transcription reaction, 1 unit of DNase I was added and the reaction was incubated for 15 min at 37°C. RNA was purified using the RNeasy MinElute Cleanup kit (Qiagen).

For synthesis of the 5′-capped p53-554 RNA, *in vitro* transcription was performed in the presence of 3 mM Anti-Reverse Cap Analogue (ARCA, Epicentre Biotechnologies), 1.5 mM GTP and 7.5 mM of the remaining nucleotide triphosphates.

### *In vitro* translation

Translation reactions were performed in nuclease-treated rabbit reticulocyte lysate (RRL) as it described in our previous work [27]. Briefly, 2.5 pmol of the uncapped p53-554 RNA or the uncapped Hp53-554 RNA, in a volume of 5.5 μl, was denatured at 65°C for 3 min and immediately placed on ice for 5 min. The RNA solution was added to 19.5 μl of the translation mixture composed of 17.5 μl of RRL, 20 μM amino acid mix without methionine, 1 μl of [^35^S]-methionine (1000 Ci/mol) (Hartman Analytic) and 20 units of recombinant ribonuclease inhibitor (Promega). The reaction mixture was incubated for 2 min at 30°C, then a specific antisense oligonucleotide was added to a final concentration of 1 μM and the reaction proceeded at 30°C for 90 min. After incubation, RNase A was added to a final concentration of 0.2 mg/ml and the samples were incubated for 5 min at room temperature. Translation reaction products were resolved on 15% SDS-polyacrylamide gels, followed by gel drying and radioisotope imaging with FLA 5100 image analyser (Fuji). Band intensities were analyzed using MultiGauge software (Fuji).

For experiments with the cap analog (m^7^GpppG) as an inhibitor, the 5′-capped p53-554 RNA was used. RRL was pre-incubated for 15 min at 30°C with an increasing concentration of the m^7^GpppG cap analog (0–750 μM; Epicentre Biotechnologies) and equimolar amounts of magnesium acetate.

### Pb^2+^-induced cleavage and primer extension

Prior to the cleavage reaction with Pb^2+^ ions, approximately 14 pmol of unlabelled p53-554 RNA was renatured in the buffer: 40 mM NaCl, 10 mM Tris-HCl pH 7.2, 10 mM MgCl_2_ by heating for 5 min at 65°C and cooling for 10 min at 37°C. Then, antisense oligomer was added to the reaction to a final concentration of 1 μM and the mixture was incubated at 37°C for 5 min. The mixture was divided into three samples and lead acetate solution was added to a final concentration of 0.5 mM or 2 mM and an equal volume of water was added to the control reaction. After incubation at 37°C for 3 min, the reactions were terminated by mixing the aliquots with 8 M urea/20 mM EDTA solution, the RNA with antisense oligomers were precipitated with 0.3 M sodium acetate pH 5.2, 1 μl of glycogen (20 mg/ml) and 3 volumes of ethanol. To determine Pb^2+^-induced cleavage sites, a primer extension reaction was performed with 5′-end-[^32^P]-labelled primer R554AUGb: 5′-TCATCTGGACCTGGGTCTTCAG-3′ or Rdlug: 5′-TCCATTGCTTGGGACGGCAAGG-3′ (for oligomer 1) at 42°C for 40 min with 100 units of RevertAid^TM^ M-MuLV reverse transcriptase (Fermentas). The Pb^2+^-induced cleavage reaction products were resolved on 8% polyacrylamide gels, followed by radioisotope imaging with FLA 5100 image analyser (Fuji).

## Results

### Binding of antisense oligonucleotides causes local structural changes in the 5'-terminal region of p53 mRNA

Five antisense oligonucleotides which were potentially able to hybridize to the 5'-terminal region of p53 mRNA were designed based on our previous work [27]. Oligomers No. 1 and No. 4 bind to the G56-C169 stem-loop containing the initiation codon AUG1 for the full-length p53, whereas oligomers No. 7, 7a and 7b are complementary to the U180-U228 region (Fig 1A) which is believed to interact with Hdm2 protein [20]. All the oligomers exhibit similar overall free energy of the RNA target-oligomer hybrids (Fig 1B) in order to prevent one of the oligomers being thermodynamically favoured in binding to the target RNA [30].

**Fig 2:**
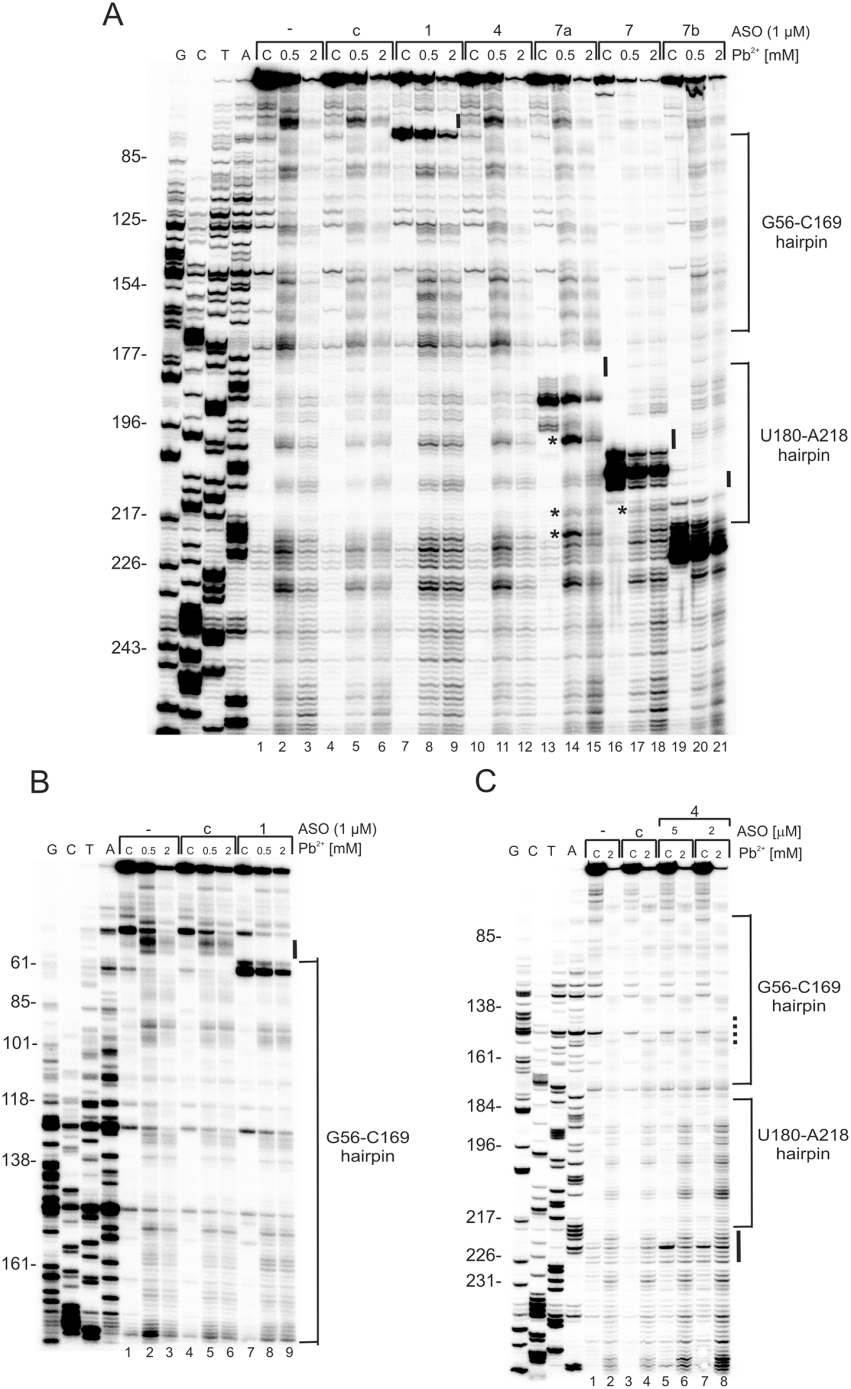
Structure probing of p53-554 RNA in the presence of specific 2′-*O*Me antisense oligomers. Autoradiograms show the products of Pb^2+^-induced cleavage analyzed by primer extension with R554AUGb **(A)** and Rdlug **(B)** primers on 8% polyacrylamide gels in denaturing conditions. Lanes 1–3: reactions in the absence of oligomer; lanes 4–6: reactions with a control oligomer; lanes 7–21: reactions with oligomers No. 1, 4, 7a, 7, 7b and the control oligomer respectively (Table 1). G, T, C, A—sequencing reactions with dideoxy terminating nucleotides. The changes in RNA structure upon oligomer binding are marked by: black line along the gel—disappearance of Pb^2+^ cleavages compared to control reactions, (A and B); asterisk (*)–presence of additional Pb^2+^ cleavage sites compared with control reactions, (A and B). **(C)** Non-specific hybridization effect of oligonucleotide No. 4. Autoradiogram shows the products of Pb^2+^-induced cleavage analyzed by primer extension with R554AUGb. G, T, C, A—sequencing reactions with dideoxy terminating nucleotides. Dotted line along the gel—binding site of oligomer No. 4 predicted *in silico*. Black line along the gel—binding sites of oligomer No. 4 detected experimentally.

In order to confirm binding of the antisense oligomers to the 5'-terminal region of p53 mRNA and reveal the structural rearrangements occurring upon the binding, the structure of this region was probed with Pb^2+^ ions *in vitro* [28, 31]. The Pb^2+^-induced cleavage sites were determined by the primer extension method. The Pb^2+^ treated RNA was precipitated with antisense oligonucleotides still present in the mixtures, which causes the reverse transcriptase to stop when it encounters the hybridized oligomers. Primer extension stops were observed on the gels in the regions corresponding to where the oligonucleotides bind (Fig 2).

The mapping results showed that oligomers No. 7 and 7b bind to the RNA with high affinity, which is reflected by strong primer extension stops (Fig 2A, lanes: 16–21). In the case of oligomer No. 7, there are no Pb^2+^ cleavage sites in the 3' side of the apical loop (198-CCUA-201) of the U180-A218 hairpin (Fig 2A, lanes: 17–18). On the other hand, with oligomer No. 7b, no Pb^2+^ cleavage sites are observed in the internal loop (208-CUAC-211) of the hairpin (Fig 2A, lanes: 20–21). Evidently, the changes in the Pb^2+^-induced cleavage patterns result from the binding of both oligomers No. 7 and 7b to the expected sites along the 5'-terminal region of p53 mRNA. Thus, these oligomers cover the single-stranded regions of the U180-A218 hairpin, which might be recognized by cellular proteins (Fig 1A). Moreover, in the case of oligomer No. 7 additional Pb^2+^ cleavages appear at the bottom of the U180-A218 hairpin (216-UGA-218) (Figs 1A and 2A, lanes 17–18; denoted by the asterisk on the autoradiogram). It seems that oligomer No. 7 partly unfolds the lower part of the hairpin stem. Binding of oligomer No. 7a to the RNA seems to be weaker than that of oligomers 7 and 7b, since no such strong primer extension stops are observed on the gel (Fig 2A, lanes 13–15). However, oligomer No. 7a causes higher susceptibility of the 3' side of the U180-A218 hairpin to Pb^2+^ cleavage, resulting in stronger cuts being observed in the apical loop (Fig 2A, lanes 14–15; the upper asterisk on the autoradiogram). Furthermore, in the case of oligomer No. 7a, additional cleavages occur at the bottom of the U180-A218 hairpin, similar to those seen for oligomer No. 7 (Fig 2A, lanes 14–15 and 17–18, the middle asterisk on the autoradiogram). We also observe strong cleavages in the A residues tract, A219-A224, compared with the control lanes (Fig 2A, lanes 14–15; the bottom asterisk on the autoradiogram; control lanes 2–3 and 5–6). Therefore, the oligomers 7, 7a and 7b, which hybridize to the U180-U228 region, all are not only responsible for local structural changes, but also affect the surroundings of their binding sites as well. However, other more remote parts of the 5' terminal region of p53 mRNA are apparently not affected, since the patterns of Pb^2+^ cleavage do not change.

Primer extension stops observed in the gel in the presence of oligomer No. 1 confirm its ability to hybridize to the expected region of the G56-C169 hairpin (Fig 2A and 2B, lanes 7–9). Moreover, no Pb^2+^ cleavage sites are present at the bottom of the hairpin and in the adjacent 5' flanking region, compared to the control reaction (Fig 2A and 2B, lanes 8–9; control lanes 2–3 and 5–6). Surprisingly, no Pb^2+^-revealed binding effects are observed for oligomer No. 4 in the expected region on the gel (Fig 2A, lanes 10–12). In an attempt to explain this observation, Pb^2+^-induced cleavage was performed at a higher final concentration of oligomer No. 4, amounting to 2 μM and 5 μM (Fig 2C). The analysis revealed that the oligomer appears to hybridize non-specifically around position 220 of the RNA chain. A possibility of a false binding site was also suggested *in silico* by the Oligo Program (data not shown).

In summary, the results of structural probing with Pb^2+^ demonstrate that all the studied oligomers except oligomer No. 4 bind to the predicted sites along the RNA target. Moreover, oligomers No. 7, 7a and 7b, which hybridize to the U180-U228 region, are able to locally modify the structure of the 5'-terminus region of p53 mRNA.

### Intact global structure of the 5'-terminal region of p53 mRNA is necessary for efficient translation of Δ40p53 in HT-29 cells under oxidative stress

**Fig 3:**
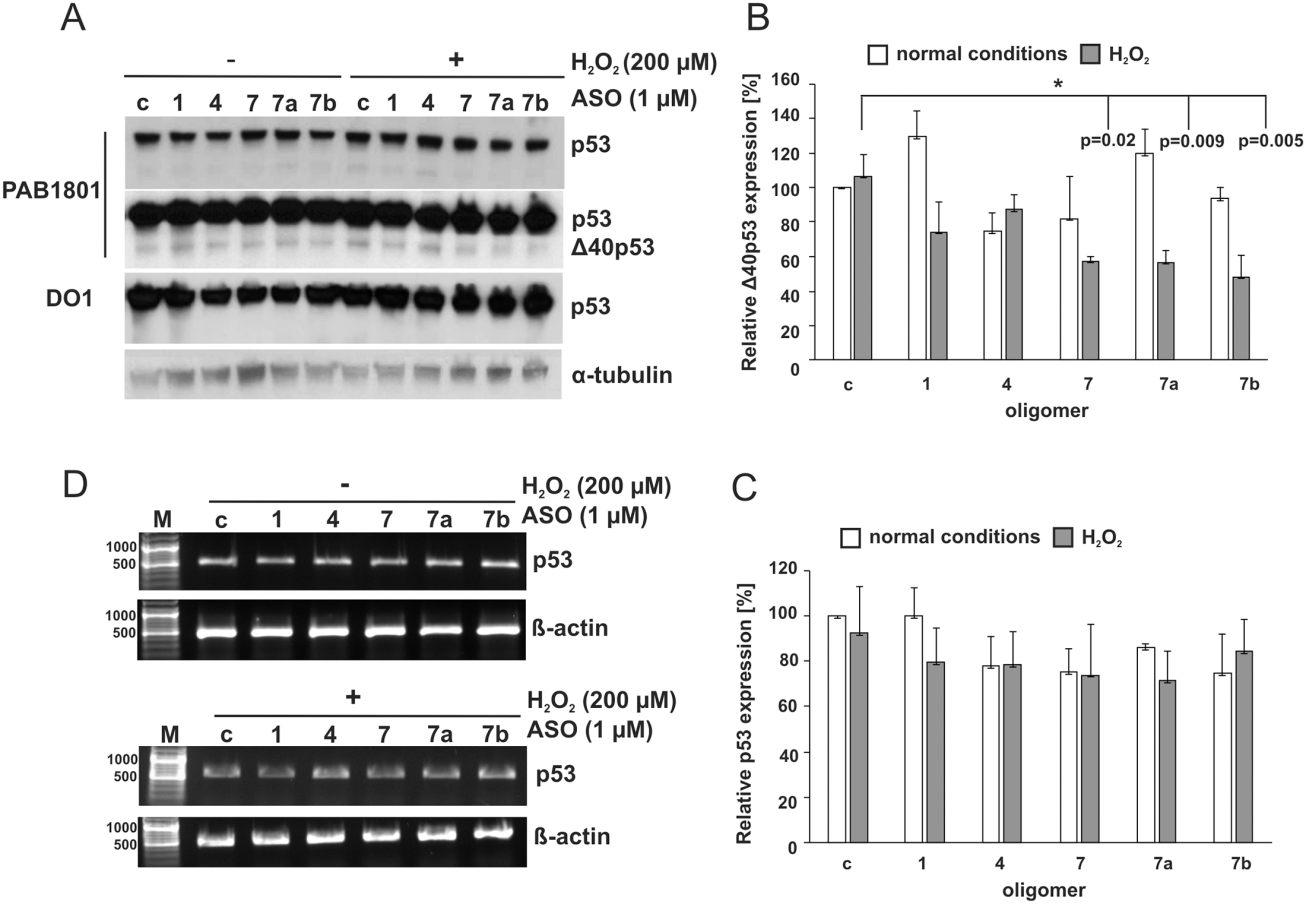
The p53 and Δ40p53 translation efficiency in the presence of antisense oligonucleotides complementary to the 5'-terminal region of p53 mRNA under conditions of with and without oxidative stress in HT-29 cells. The cells were transfected with oligomers No. 1, 4, 7, 7a, 7b, and the control oligomer, respectively. Subsequently, the cells were exposed to stress conditions for 6 hours and then harvested. **(A)** Endogenous p53 and Δ40p53 level was determined by Western blot using two specific monoclonal antibodies p53: Pab 1801 (two panels are shown—with short and long exposures of the gel) and DO1. The long exposure revealed lower band, corresponding to the Δ40p53 isoform. The α-tubulin level was used as a loading control. **(B)** The Δ40p53 level in the presence of selected 2′-*O*Me oligomers was compared to the value obtained with control oligomer under normal conditions, which was defined as 100%. **(C)** The p53 level in the presence of selected 2′-*O*Me oligomers was compared to the value obtained with control oligomer under normal conditions, which was defined as 100%. The bar graphs show averages and standard deviations for at least three independent experiments. White and grey bars indicate normal and stress conditions, respectively. (*) p-values were calculated using Student’s t-test. **(D)** The RT-PCR analysis of p53 mRNA and β-actin mRNA (as a control) extracted from cells after transfection with 2′-*O*Me oligomers and incubation of the cells in the absence; (-) and the presence (+) of hydrogen peroxide. At least two independent experiments were done to verify that no changes in the p53 mRNA level takes place.

To evaluate the role of the 5'-terminal region of p53 mRNA in the translation initiation process under stress conditions selected antisense oligomers were applied in the HT-29 colorectal adenocarcinoma cell line. These cells contain a G to A point mutation in codon 273 of the *p53* gene, which results in an arginine—histidine substitution (R273H). This mutation results in an overexpression of p53 protein [32]. The employed oligomers bore methyl groups at the 2' hydroxyl positions of each of the ribose residues to prevent triggering the RNase H activity present in the cells and subsequent degradation of the p53 mRNA [29]. This excluded RNase H-dependent effects from analysis. Additionally, we previously showed that p53 mRNA levels were unchanged even after 10 hours after 2′-*O*Me oligomer transfection [27]. This indicates that 2′-*O*Me oligomer may disturb the translation process *via* structurally disordering the RNA, but RNA transcription and stability are unaffected. The level of p53 synthesis in the presence of antisense oligonucleotides was analysed under normal conditions and conditions of oxidative stress. Hydrogen peroxide, at a final concentration of 200 μM, was added to the cell culture 4 hours post-transfection and the cells were subsequently incubated for additional 6 hours. The monoclonal antibody PAB1801, which recognizes amino acid residues 46–55 of the N-terminal region of p53, revealed two bands on a Western blot (Fig 3A, two panels show short and long exposure of the autoradiogram). The major band corresponds to the full-length p53 protein, while the minor one is its isoform, with a molecular weight of approx. 44–46 kDa. The monoclonal antibody DO1 only detected the upper band on the gel (Fig 3A). This antibody only recognizes FLp53 and its isoforms which have the entire N-terminal region, since the antibody is directed against amino acid residues 21–25 of the N-terminus of the p53 protein [18]. It has been previously shown that in HT-29 cells not only FLp53, but also its isoforms, can be detected by Western blotting using the polyclonal antibody CM1 [2]. Thus, most likely, the lower migrating band on the gel, detected by PAB1801 but not detected by DO1, corresponds to the Δ40p53 isoform.

Unexpectedly, none of the antisense oligomers tested substantially affected the translation of the full-length p53 under the conditions applied (Fig 3A and 3C). The lack of effect of the oligomers might be at least partially explained by the overproduction of the p53 protein in HT-29 cells. On the other hand, all the applied antisense oligonucleotides impair the synthesis of Δ40p53 isoform under conditions of stress. A strong decrease of the Δ40p53 level, by approximately 50%, was observed for oligomers No. 7, 7a and 7b, all of which hybridized to the U180-U228 region (Figs 1A and 3B). Synthesis of Δ40p53 was also reduced by approx. 20% in the presence of oligomer No. 1. Despite the fact that hybridization of oligomer No. 4 to the RNA target *in vitro* was non-specific and weak, it reduced the efficiency of Δ40p53 translation by approximately 10% (Fig 3B). Oligomer No. 4 seems to be able to weakly bind to the U180-A218 hairpin, which may explain this observation. In the absence of hydrogen peroxide-induced stress, three out of five tested oligomers only slightly reduced the level of the Δ40p53 isoform (Fig 3B).

To confirm that RNase H was not involved in the observed reduction of the Δ40p53 protein level, RT-PCR analysis was performed (Fig 3D). The level of p53 mRNA was unchanged in the presence of oligomers under normal and stress conditions.

It has been proposed that the Δ40p53 isoform can be produced in a cap-independent manner [14, 18]. Additionally, overall cap-mediated translation has been shown to be impaired under conditions of stress [33]. Since the expression of Δ40p53 is inhibited in the presence of the tested oligomers under oxidative stress, it suggests that IRES-mediated translation is affected. Moreover, the entire structure of the 5'-terminus of p53 mRNA seems to be important for translation efficiency of Δ40p53 in the HT-29 cells. However, the translation initiation is particularly sensitive to any disturbance of the structure of the U180-U228 region.

### Remodelling of the U180-A218 hairpin upon oligomer hybridization influences translation efficiency of FLp53 protein in MCF-7 cells under genotoxic and oxidative stress

**Fig 4:**
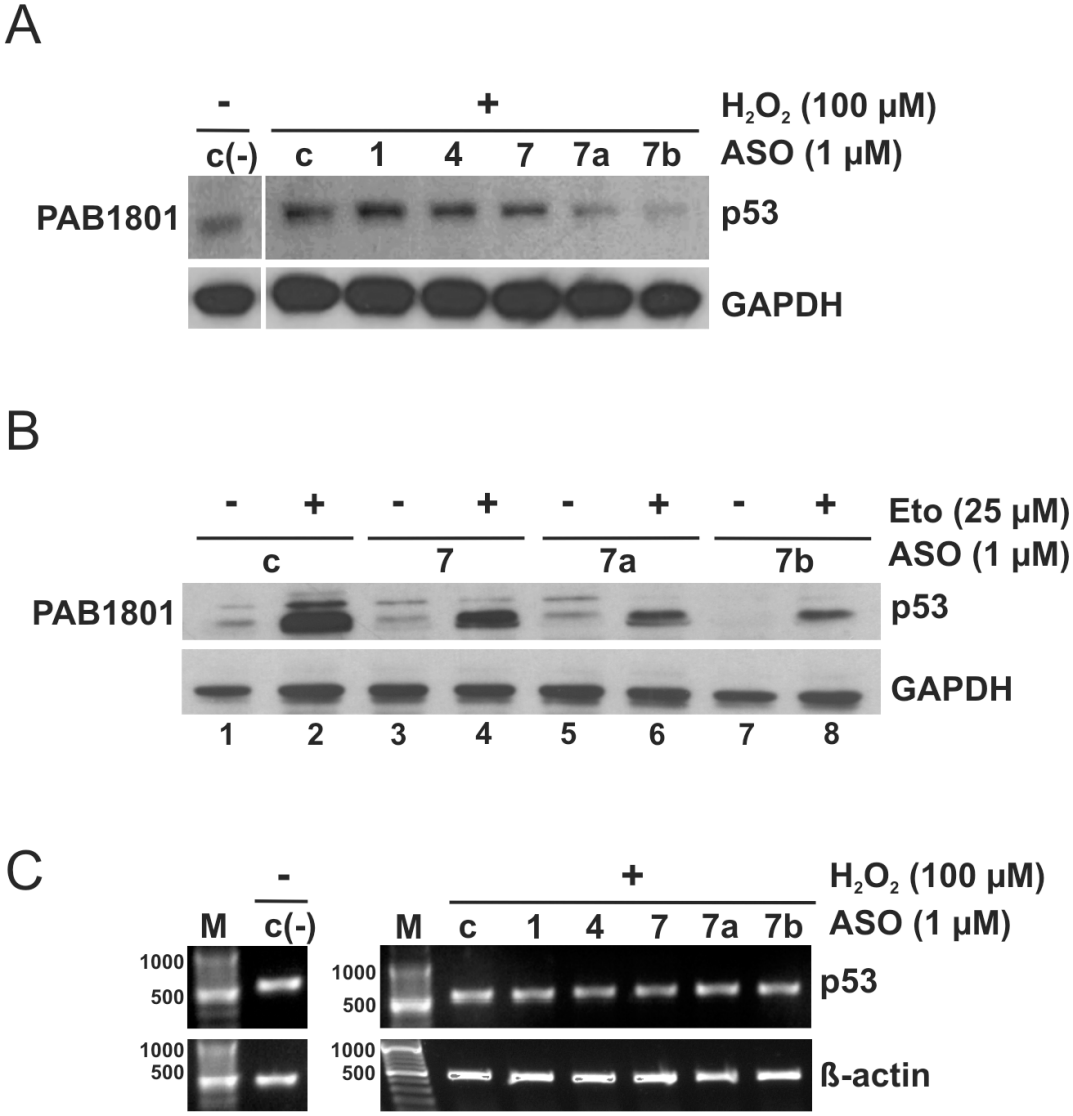
Impact of antisense oligomers hybridized to the U180-A218 hairpin on FLp53 translation in MCF-7 cells under conditions of oxidative and genotoxic stress. After oligomer transfection the cells were treated with **(A)** hydrogen peroxide at a final concentration of 100 μM and **(B)** 25 μM etoposide. Western blot analysis was performed with a monoclonal antibody (Pab 1801) to detect p53 levels. The GAPDH level was used as a loading control. The experiments were repeated at least twice. Representative Western blots are displayed in the figure. **(C)** The RT-PCR analysis of p53 mRNA and β-actin mRNA (as a control) extracted from the cells after 2′-*O*Me oligomers transfection and incubation the cells in the absence; c(-) and the presence (+) of hydrogen peroxide, respectively. At least two independent experiments were performed, which showed no changes in p53 mRNA level.

To elucidate which structural elements of the 5'-terminal region of p53 mRNA are important for FLp53 translation efficiency in the presence of selected stress factors, the antisense oligonucleotides were applied to MCF-7 breast carcinoma cells. The cells were exposed to 100 μM hydrogen peroxide after oligomer transfection, and subsequently, incubated for 6 hours. The antibody Pab1801 detected only one band, corresponding to FLp53 (Fig 4A). This is consistent with previously published data demonstrating a lack of Δ40p53 detection/activation upon oxidative stress in MCF-7 cells [11]. Hydrogen peroxide caused an elevation of the FLp53 protein level, as expected (Fig 4A, lanes c and c(-)). In the presence of oligomers No. 7a and 7b, hybridizing to the U180-A218 hairpin and the A219-U228 single-stranded stretch, respectively, decreases in FLp53 translation were observed. Slight translation inhibition was also observed with oligomer No. 7. In contrast oligomers No. 1 and 4 barely affected the efficiency of the FLp53 synthesis (Fig 4A). RT-PCR analysis showed no changes in the level of p53 mRNA in the presence of 2′-*O*Me oligomers (Fig 4C).

To examine whether our observations can be generalized to other stress-inducing agents, we applied oligomers No. 7, 7a and 7b upon genotoxic stress conditions generated in MCF-7 cells by etoposide at a final concentration of 25 μM. In the absence of a stressor, essentially only oligomer No. 7b inhibited translation of the FLp53 (Fig 4B, lane 7), similar to what we have observed in the earlier studies [27]. Under conditions of stress, the p53 protein level was much higher compared with non-treated cells, as expected (Fig 4B, lanes 1, 2). Oligomers No. 7, 7a and 7b caused a decrease in the level of FLp53 translation and the inhibitory effect was most striking for oligomer No. 7b. (Fig 4B).

Taken together, under oxidative and genotoxic stress the translation efficiency of FLp53 strongly depends on the presence of the intact U180-A218 motif and the adjacent A219-A228 single-stranded stretch in the 5'-terminal region of p53 mRNA. The antisense oligomer effects seem to be similar under both kinds of stress. Since the stimulation of translation occurs more effectively with Eto (etoposide), in consequence, in the presence of the best performing oligomer 7b the translation efficiency is higher in Eto and lower in H_2_O_2_ when compared to the control without a stressor.

### The FLp53 and Δ40p53 level is modulated by binding of oligomers to the U180-U228 region under stress conditions in HepG2 cells

**Fig 5:**
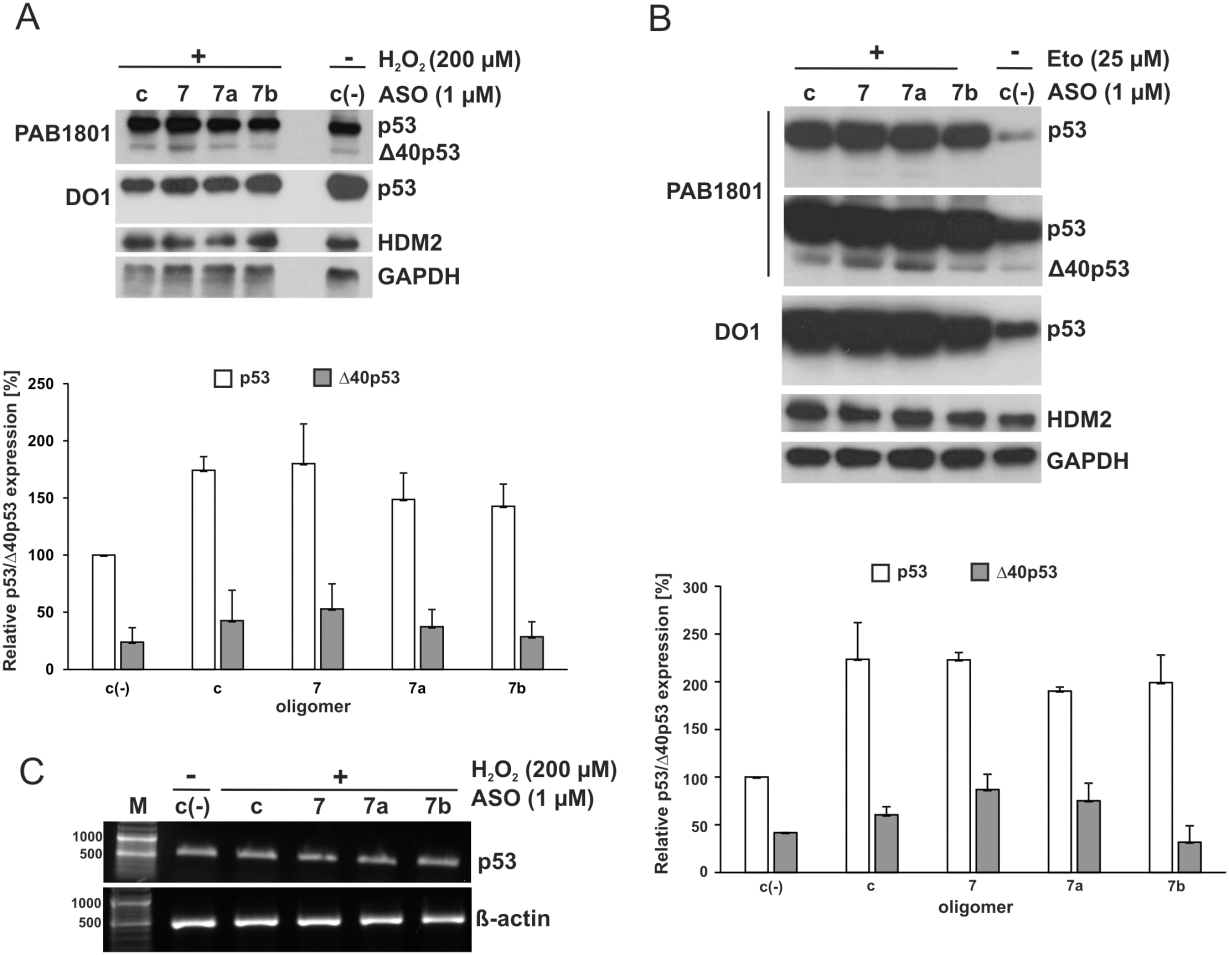
Modulation of the p53 and Δ40p53 translation initiation process by antisense oligomers complementary to the U180-U228 region in HepG2 upon stress conditions. Cell transfection with oligomers No. 7, 7a, 7b, and control oligomer followed by **(A)** oxidative and **(B)** genotoxic treatment, respectively. Endogenous p53, Δ40p53 and Hdm2 proteins’ level was analyzed by Western blot as described in Fig 3. The GAPDH level was used to normalize the data. The levels of p53 and Δ40p53 proteins in the presence of selected 2′-*O*Me oligomers were compared to the value obtained with the control oligomer under normal conditions, which was defined as 100%. The bar graphs show averages and standard deviations for at least three independent experiments. **(C)** The RT-PCR analysis of p53 mRNA and β-actin mRNA (as a control) extracted from the cells after 2′-*O*Me oligomers transfection and incubation the cells in the absence; c(-) and the presence (+) of hydrogen peroxide, respectively. At least two independent experiments were performed.

We showed that the antisense oligonucleotides affected either FLp53 in MCF-7 cells, or Δ40p53 in HT-29 cells, in which both forms of p53 protein are present, but FLp53 is mutated and overexpressed. We were curious to know whether the oligomers were able to simultaneously affect both of the wild-type p53 isoforms. Antisense oligomers No. 7, 7a and 7b targeting the U180-U228 region were applied in hepatocellular carcinoma cells, HepG2, in which the wild type FLp53 and its Δ40p53 isoform are expressed under normal conditions [11]. The presence of both isoforms of p53 was confirmed by PAB1801 and DO1 antibodies (Fig 5). Under oxidative stress, only modest effects of the oligomers on the translation were observed (Fig 5A). In order to make sure that this was not due to inefficient oligomer incorporation into the cell, a DNA oligomer labelled with fluorescein (FAM) was applied in our assay. Transfection of FAM-conjugated oligomer proved no limitation at this stage of the experiment (data not shown). We observed that under oxidative stress, oligomer No. 7b was most effective in inhibiting both FLp53 and Δ40p53 synthesis by about 10–20%. No change in Hdm2 levels occurred (Fig 5A).

Subsequently, antisense oligomers No. 7, 7a and 7b were applied in HepG2 cells in the presence of etoposide, a genotoxic factor. Little effect of the oligomers on the translation of FLp53 was observed (Fig 5B). Yet the synthesis of Δ40p53 was impaired by about 40% with oligomer No. 7b, which also caused strong inhibition of FLp53 translation in MCF-7 treated with etoposide (Fig 4B). Surprisingly, oligomers No. 7 and 7a increased the Δ40p53 level by approximately 25–30% (Fig 5B). In the presence of these oligomers a slight increase in the Δ40p53 level was also observed upon oxidative stress conditions (Fig 5A). It is worth noting that structural probing of the 5'-terminus of p53 mRNA in the presence of oligomers No. 7 and 7a revealed partial unfolding of the U180-A218 hairpin (Fig 2A). Similar to the observations in HT-29 and MCF-7 cells, no alterations in p53 mRNA level were detected in the presence of oligomers under normal and stressed conditions (Fig 5C).

We speculate that in the case of HepG2 treated with etoposide, hybridization of oligomers facilitates translation initiation by exposing the p53 mRNA fragment, which may interact with specific ITAFs. Changes in the Δ40p53 level were not reflected in modulation of the amount of Hdm2 (Fig 5B). Surprisingly, our data showed that contrary to the inhibitory effects observed on p53 synthesis in MCF-7 cells under genotoxic stress, in HepG2 cells only a slight decrease of FLp53 and increase of Δ40p53 synthesis was observed in the presence of specific oligonucleotides. These observations might suggest different structural RNA requirements for translation efficiency of FLp53 and its isoform in various cell types upon stress conditions. However, we cannot exclude other scenarios, such as different proteins’ influence on the p53 and Δ40p53 synthesis in MCF-7 and HepG2 cell cultures.

### Structural features of the 5'-terminus of p53 mRNA required for translation from the initiation codons AUG1 and AUG2 in rabbit reticulocyte lysates

We decided to compare the results from the analysis of living cells with the data obtained from *in vitro* analysis of the translation initiation process in rabbit reticulocyte lysates (RRL). The cell lysate is a simplified model system which lacks some of the proteins present in living cells. Therefore, it facilitates the evaluation of the impact of the RNA structure on translation.

**Fig 6:**
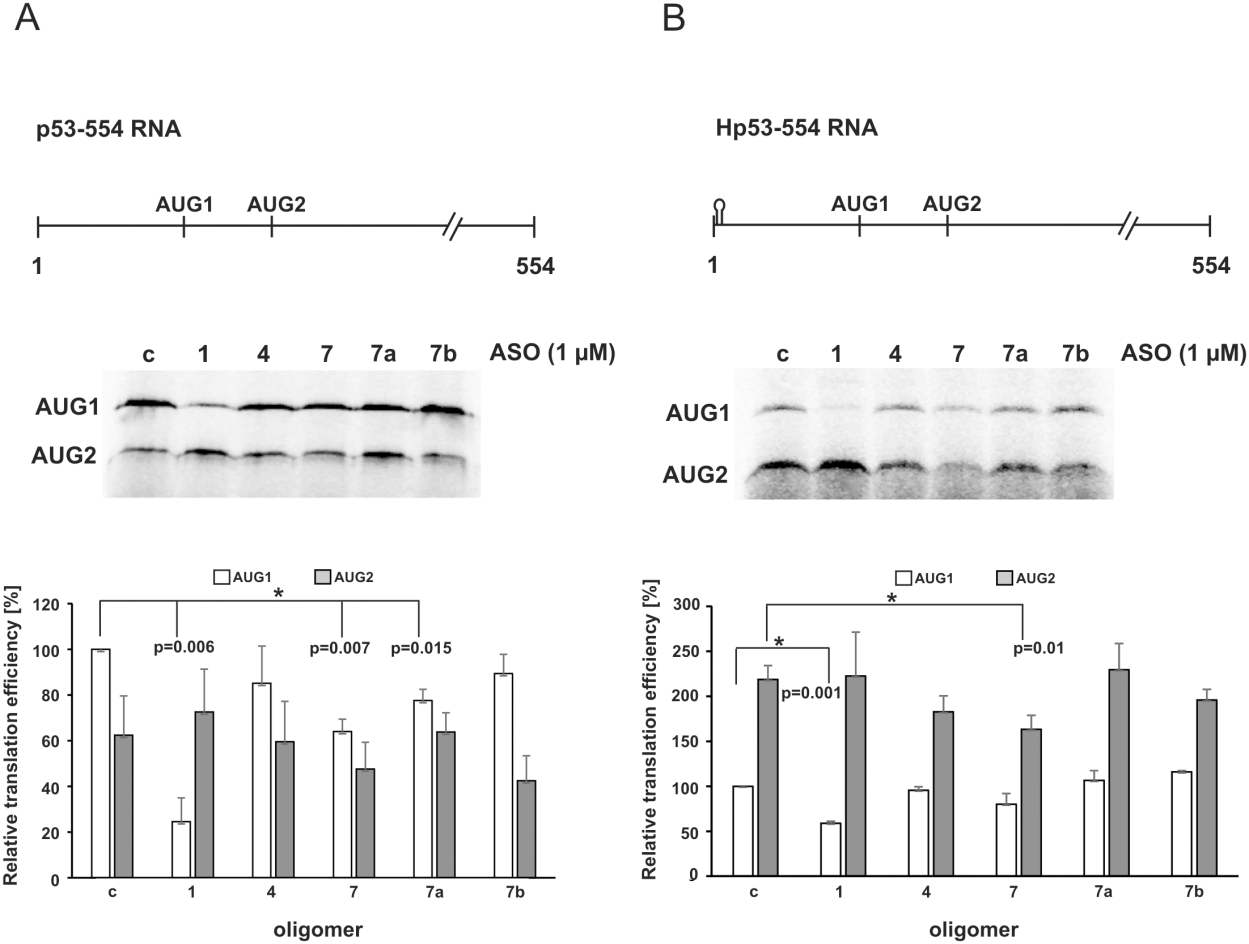
Impact of antisense oligomers on translation initiation from AUG1 for p53 and from AUG2 for Δ40p53, in rabbit reticulocyte lysates. **(A)** Translation of the uncapped p53-554 RNA and **(B)** The uncapped Hp53-554 RNA constructs in the presence of specific oligomers No. 1, 4, 7, 7a, 7b and the control oligomer, respectively. The autoradiograms show the translation products from both initiation codons, AUG1 and AUG2, with p53-554 RNA (A) and Hp53-554 RNA (B). The bar graphs display translation efficiency for AUG1 and AUG2 codons with p53-554 RNA (A) and Hp53-554 RNA (B). All values are averages of at least three independent experiments and were normalized to the translation efficiency of product from AUG1 for p53. (*) p-values were calculated using Student’s t-test.

It is known that the lack of the cap structure at the 5' end of mRNA impairs the recognition of mRNA by the ribosomal initiation complex, and as a consequence cap-mediated translation is affected [34]. It has also been shown that a thermodynamically stable hairpin at the 5'-terminus of both capped and uncapped mRNA template can inhibit ribosome scanning of mRNA [19]. Thus, alternative translation mechanisms of protein synthesis can be easily imagined [18]. Recently, it has been demonstrated that the presence of a stable hairpin at the 5' end of the uncapped RNA template corresponding to 554 nucleotides of the 5'-terminal region of p53 mRNA caused inhibition of the translation efficiency from AUG1 for FLp53. Moreover, a strong increase in protein synthesis from AUG2 for Δ40p53 was observed [19]. To establish the antisense oligomer effects on IRES-mediated translation, two uncapped transcripts of p53 mRNA, one with an additional hairpin at the 5' end, were used (Fig 6).

*In vitro* translation assays using a control oligonucleotide but no test oligonucleotide, showed an AUG1/AUG2 ratio of approx. 2:1 with p53-554 RNA and approx. 1:2.5 with Hp53-554 RNA, which is consistent with the previous data (Fig 6A and 6B, lanes c and reference [19]). Oligomer No. 1 complementary to the G56-C169 motif significantly inhibited translation from AUG1 with both RNA constructs, whereas the effect of the other oligomers was smaller (Fig 6A and 6B). This might be explained by low helicase activity in the cell lysate resulting in difficulties in unwinding the long helical structure of G56-C169 with the hybridized antisense oligomer. Since the translation levels of neither mutant nor wild type FLp53 were affected by oligomer No. 1 in the cells (Figs 3 and 4) the oligomer probably does not compete with cellular proteins for the binding site to the RNA. Presumably, it does not cause any significant RNA structural rearrangements in the cell either. We did not observe any additional structural changes upon hybridization of oligomer No. 1 *in vitro*, other than the disappearance of Pb^2+^ cleavage sites in its binding site (Fig 1B and 1C, lanes 8–9). Another possible explanation of such strong inhibitory effects occurring upon binding of oligomer No. 1 is disturbance of the IRES—mediated translation of p53. It has been proposed that translation from both codons: AUG1 and AUG2, is governed by IRES element(s) [35]. Unexpectedly, the inhibitory effect of oligomer No. 1 is not observed in the cells we tested (Figs 3 and 4). We speculate that its impact may be minimized *in vivo* due to the presence of additional protein factors, ITAFs, which facilitate IRES-dependent synthesis of FLp53.

Oligomer No. 7 inhibited protein synthesis initiated from AUG1 with p53-554 RNA by about 35%, and with Hp53-554 RNA by about 20% (Fig 6A and 6B). With p53-554 RNA, the synthesis from AUG1 was also impaired by oligomer No. 7a by approx. 20% (Fig 6A). These results correspond to the observations made in the MCF-7 cells. Oligomers No. 7 and 7a inhibited the translation efficiency of FLp53 in these cells under different stress conditions (Fig 4). However, no influence was observed in the presence of oligomer No. 7b *in vitro*, despite its strong effect in living cells (Figs 4 and 5).

Translation from AUG2 with the p53-554 RNA template was impaired by approximately 30% with oligomer No. 7b and by about 15% with oligomer No. 7 (Fig 6A). A stronger inhibitory effect of oligomer No. 7 on the synthesis from AUG2, involving an approximately 25% decrease of the translation efficiency, was observed with Hp53-554 RNA (Fig 6B). However, no changes occurred in the presence of oligomer No. 7a using either RNA template (Fig 6). Interestingly, in HepG2 cells, upon genotoxic stress a stimulatory effect of oligomer No. 7a was demonstrated (Fig 5B). Oligomer No. 4 reduced protein synthesis from AUG2 *in vitro* with Hp53-554 RNA only by approx. 15%, similarly to the effect observed in HT-29 cells (Figs 3 and 6B).

**Fig 7:**
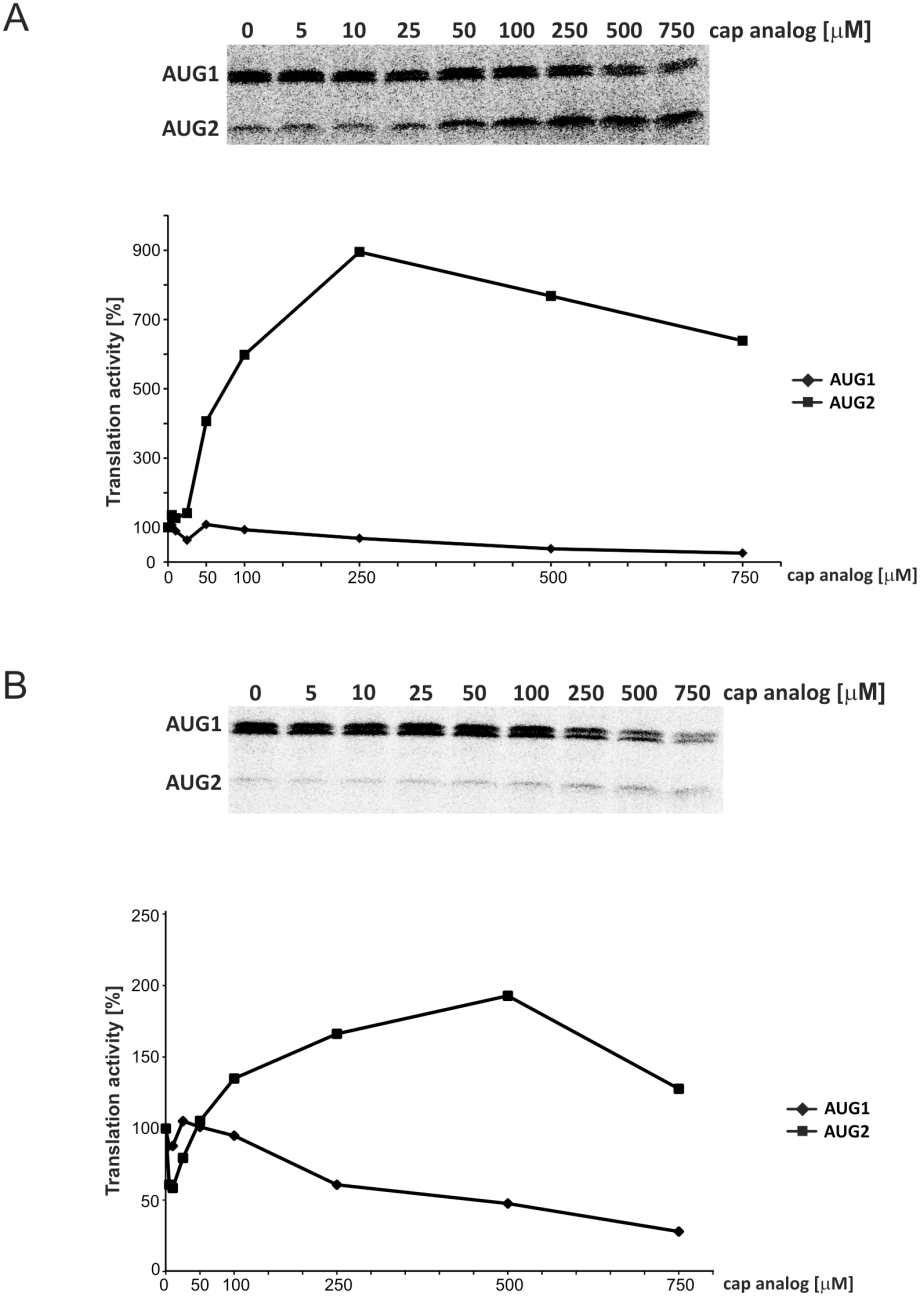
*In vitro* translation of the p53-554 RNA in RRL in the presence of antisense oligomers No. 1 (A) and no. 7 (B) used at a concentration of 1 μM upon increasing the concentration of the cap analog (m^7^GpppG) to inhibit cap-dependent translation. The 5′-capped p53-554 RNA was translated in RRL in the presence of ^35^S-methionine and relative amounts of translation products from initiation codons AUG1 and AUG2 were determined. Following normalization to the values with no cap analog added, they were displayed in the figure. The quantitative data presented in panels A and B were calculated based on at least three independent experiments.

Finally, the 5′-capped p53-554 RNA was translated in the presence of oligomers No. 1 and No. 7, and an increased concentration of cap analog (m^7^GpppG) to inhibit cap-dependent translation (Fig 7). In the case of both antisense oligomers, translation from AUG1 was inhibited by the addition of the cap analog. The changes were relatively small up to an analog concentration of 100 μM and the translation efficiency did not exceed 30% of the control value at a concentration of 750 μM of the cap analog. In contrast, translation from AUG2 showed a strong dose-dependent increase and at maximum the translation activity exceeded 900% and 200% of the reaction control in the presence of oligomers No. 1 and 7, respectively (Fig 7).

The translation assays performed in RRL suggest that structural features of the U180-U228 region of the 5'-terminus of p53 mRNA are important for the efficiency of translation from AUG1 (Fig 6A) and AUG2 (Fig 7A and 7B). In addition, an intact G56-C169 hairpin is crucial to maintain high efficacy of translation in the cell lysate. However, comparing the oligomers’ effect in RRL and in the cell cultures, we observed some discrepancies. They may result from “incomplete” proteins’ composition in the lysate compared to that in living cells. Nevertheless, the data obtained *in vitro* showed how structural disturbances of the 5' end of p53 mRNA could affect translation from AUG1 for FLp53 and AUG2 for Δ40p53.

## Discussion

It has been suggested that the translation initiation process of full-length p53 and its *N*-truncated isoform Δ40p53 are driven by an IRES element present in the 5'-terminal region of p53 mRNA [14, 17, 18]. The multiple alignment of RNA sequences of this region composed of the 5'-untranslated part and 40 amino acids of FLp53 coding sequence revealed that it is conserved among mammalian cells [19]. On the basis of the information provided by the p53 database we know that in tumor cells there are far fewer mutations in the 5'-terminal region of p53 mRNA compared to the downstream coding sequence [36]. Moreover, SNPs or mutations which have been identified in this region are often correlated with a lower rate of p53 synthesis and disturbance of its function [20, 23]. The observed strong sequence conservation of the 5'-terminus of p53 mRNA suggests that it is required to maintain the proper RNA folding crucial to the function of this region. This concept has been supported by the results of the IRES-dependent translation of viral RNAs and it has also been postulated for cellular IRES activity [21, 22, 37–39].

In order to obtain more information on the role of the specific structural motifs of the 5'-terminus of p53 mRNA in translation we used an antisense approach. We found that the unperturbed U180-U228 region is required for high translation efficiency, particularly under conditions of stress. Antisense oligomers No. 7, 7a and 7b locally misshaped the U180-A228 region, causing a decrease in the efficiency of the FLp53 or/and Δ40p53 translation (Figs 3–5). Oligomers No. 7, 7a, and part of oligomer No. 7b, bind to the U180-A218 hairpin. The hairpin has been shown to interact with several proteins, which play the role of ITAF factors, modulating the IRES activity [37]. Earlier studies on p53 mRNA mutants have revealed that a naturally occurring synonymous mutation L22L (codon in position 22, CUA to CUG), which is located in the apical loop of hairpin U180-A218, impairs p53 and Δ40p53 synthesis. This results from the disruption of the hairpin-Hdm2 interaction. The L22L mutation has been proposed to affect the tertiary structure of the U180-A218 hairpin in its apical loop region [20]. This has been revealed by stronger RNase T1 cleavages at positions G200 and G203 compared with those observed for the wild type p53 mRNA [40].

In our studies we observed that oligomer No. 7a caused a higher exposure of the 3' side of the apical loop of hairpin U180-A218 to Pb^2+^-induced cleavage. This was accompanied by a decrease in the synthesis of FLp53 in MCF-7 and Δ40p53 in HT-29 cells under conditions of stress (Figs 2B, 3B and 4). An opposite structural effect was shown with oligomer No. 7, which covered the apical loop sequence, and for which no Pb^2+^ cleavage sites were present at the 3' side of the loop (Fig 2A). Nevertheless, the translation of FLp53 and Δ40p53 was also reduced, yet to a lower extent compared to the effect caused by oligomer No. 7a (Figs 3 and 4). Thus, any structural disturbance of the apical loop of the hairpin, particularly on its 3' side, seems to affect the interaction with ITAFs, which may act as a positive regulator of the p53/Δ40p53 IRES activity. However, we cannot exclude additional superimposed effects since the binding of oligomer No. 7 or 7a also affects the stem region of hairpin U180-A218 (Fig 2A).

It has been demonstrated that remodelling of the central part of the U180-A218 hairpin in TriM p53 mRNA (with silent mutations in codons 17, 18 and 19) is probably responsible for the changes in IRES interactions with Hdm2 and hnRNP C1/C2 [20, 40]. Nevertheless, we envisage that structural alterations of the U180-A218 region might affect the interactions with other factors as well. Recently, it has been proposed that PTB binds to the A189-U194 stem region, which is rich in pyrimidines [23]. This is exactly the hybridization site of oligomer No. 7a. Thus, our results suggest that in addition to causing structural disorder, the oligomer may compete for the same binding site with PTB, which has been shown to stimulate the IRES activity [23, 40]. Thus the preservation of the entire secondary structure of the U180-A218 region seems to be required to induce IRES activity *via* interactions with ITAF factors.

Interestingly, we noticed that in the presence of oligomer No. 7b the p53 and Δ40p53 synthesis was significantly impaired not only under stressful conditions but also in the absence of stressors, consistent with our previous observations [27]. Unexpectedly, no such effects were observed *in vitro*, in rabbit reticulocyte lysates (Fig 6). Oligomer No. 7b binds mostly to the A219-U228 single-stranded stretch, which so far has not been considered a potential protein binding site. This RNA region may also interact with some non-coding RNAs inducing RNA-switching and influencing p53 translation initiation. Similarly, as it has been proposed for the 5’ untranslated region of HCV which switches from a locked to an open conformation triggered by the liver-specific microRNA, miR-122 [41]. Thus, we speculate that oligomer No. 7b might compete with auxiliary factors which are indispensable for both cap- and IRES-mediated p53 translation initiation in the living cell. However, such factors might not be necessary or even not to be present in a simplified translation system such as the cell lysates. This might explain different oligomer binding effects observed in the cell cultures compared to those in RRL (Figs 4, 5 and 6).

It is worth emphasizing that antisense oligomer effects varied between cell types, depending also on stress conditions. The presence of oligomers No. 7 and 7a augmented the Δ40p53 protein level in HepG2 cells under genotoxic and oxidative stress (Fig 5). At the same time, these oligomers caused a decrease in the Δ40p53 translation in HT-29 cells in the presence of hydrogen peroxide. Moreover, the oligomers reduced the FLp53 protein synthesis under both applied stress conditions in MCF-7 cells (Figs 3 and 4). Such differences might result from a cell-specific response to diverse stress factors [18]. Since oligomers No. 7 and 7a caused similar refolding of the lower part of hairpin U180-A218, presumably, their presence supports binding of specific proteins in HepG2 cells upon genotoxic conditions. Moreover, the effects of antisense oligomers revealed the possible regulatory mechanism of the p53/Δ40p53 ratio under various stress based on modulation of the IRES activity.

Moderate modulation effect on the translation occurred in the presence of oligomer No. 1 complementary to the G56-C169 hairpin. There was only a modest inhibition of Δ40p53 synthesis in HT-29 cells and no influence on the FLp53 amount in MCF-7 cells upon oxidative stress (Figs 3B and 4A). Markedly lower efficiency of the FLp53 translation was only observed in rabbit reticulocyte lysates (Fig 6). Recently, it has been reported that a naturally occurring C to T single-nucleotide polymorphism at position 119 in the 5'-terminus of p53 mRNA in human melanoma tumor can impair cap-independent translation. This has been explained by weaker interaction of mutated IRES with the PTB protein [23]. A comparison of the p53 translation effectiveness of the length variants of the 5'-terminal region of p53 mRNA has shown that its untranslated fragment is able to improve p53 translation. It has been associated with the binding of ribosomal protein L26 (RPL26) to this region of the mRNA, mostly under DNA damage conditions [24]. Additionally, the interaction with RPL26 has been proposed to require base pairing between the G82-G102 region (residues -54 to -34, numbering from AUG1) and a complementary fragment in the 3' UTR [42]. All these observations point to an important regulatory role of the G56-C169 motif. Additionally, this long stable hairpin with structurally disordered segments might be important for ribosome docking and interactions with translation initiation factors, similar to those observed in the case of viral IRESs [43].

We have previously shown that translation initiation from AUG2 is largely cap-independent. In particular, blocking of cap-dependent translation from AUG1 by the stable hairpin at the template 5' end did not change the level of initiation from AUG2, and upon addition of the cap analog, translation from AUG2 was increased, suggesting the presence of an IRES [19]. An increase in the translation of mRNA in the presence of cap analog implies the possibility of competition between the cap structure and the IRES for the translational machinery. Thus, the extremely strong effect observed in our current studies in the translation of p53-554 RNA in the presence of antisense oligomers No. 1 and 7 upon cap analog addition strongly supports the IRES activity located in the 5’-terminal region of p53 mRNA.

In summary, by applying the antisense oligomer approach, we were able to show that the structure of the 5'-terminus of p53 mRNA is important for the p53 and Δ40p53 translation initiation in the living cell. In particular, under conditions of stress, the U180-A218 hairpin plays a role of the *cis*-acting element which enables modulation of the IRES activity *via* interactions with protein factors. However, the p53 control mechanism at the level of translational is still a new area to be explored and further research is necessary to better understand this sophisticated regulatory system.
